# Energy Expenditure at Rest and during Walking in Patients with Chronic Respiratory Failure: A Prospective Two-Phase Case-Control Study

**DOI:** 10.1371/journal.pone.0023770

**Published:** 2011-08-31

**Authors:** Ernesto Crisafulli, Claudio Beneventi, Veronica Bortolotti, Nicoletta Kidonias, Leonardo M. Fabbri, Alfredo Chetta, Enrico M. Clini

**Affiliations:** 1 Department of Pulmonary Rehabilitation, Ospedale Villa Pineta, Pavullo nel Frignano, Modena, Italy; 2 Department of Oncology, Haematology and Pneumology, University of Modena, Modena, Italy; 3 Department of Clinical Sciences, University of Parma, Parma, Italy; Ludwig-Maximilians-Universität München, Germany

## Abstract

**Background:**

Measurements of Energy Expenditure (EE) at rest (REE) and during physical activities are increasing in interest in chronic patients. In this study we aimed at evaluating the validity/reliability of the SenseWear®Armband (SWA) device in terms of REE and EE during assisted walking in Chronic Respiratory Failure (CRF) patients receiving long-term oxygen therapy (LTOT).

**Methodology/Principal Findings:**

In a two-phase prospective protocol we studied 40 severe patients and 35 age-matched healthy controls. In phase-1 we determined the validity and repeatability of REE measured by SWA (REEa) in comparison with standard calorimetry (REEc). In phase-2 we then assessed EE and Metabolic Equivalents-METs by SWA during the 6-minute walking test while breathing oxygen in both assisted (*Aid*) or unassisted (*No-Aid*) modalities. When compared with REEc, REEa was slightly lower in patients (1351±169 *vs* 1413±194 kcal/day respectively, p<0.05), and less repeatable than in healthy controls (0.14 and 0.43 coefficient respectively). COPD patients with CRF patients reported a significant gain with *Aid* as compared with *No-Aid* modality in terms of meters walked, perceived symptoms and EE.

**Conclusions/Significance:**

SWA provides a feasible and valid method to assess the energy expenditure in CRF patients on LTOT, and it shows that aided walking results in a substantial energy saving in this population.

## Introduction

Among new methods to objectively assess Energy Expenditure (EE) both at rest (REE) or during activity, the SenseWear®Armband (SWA) device has been validated in healthy normal-weight [1]–[5] or obese [6] individuals and in chronic patients with heart failure [7] or diabetes [8].

This is a multisensor device easily applied and carried by humans that allows to continuously measure active movements and to derive energy expenditure throughout; a specific software is associated for this calculation.EE measurement by SWA has been shown to have elevated reliability during standard walked distance (6MWT) even in patients with chronic obstructive pulmonary disease (COPD) [9]–[10]. However, it is not yet proved that SWA is also feasible to assess EE during walking in those COPD patients suffering from Chronic Respiratory Failure (CRF) and requiring long-term oxygen therapy (LTOT).

These patients generally suffer from progressive physical disability and muscles de-conditioning which often cause restriction in daily activities (such as self-directed walking) [11]. In this population, a walking aid to carry oxygen canister can help to improve exercise performance and related symptoms [12]; notwithstanding, it is not known whether this improvement is also favourable in terms of EE.

## Methods

**Figure 1:**
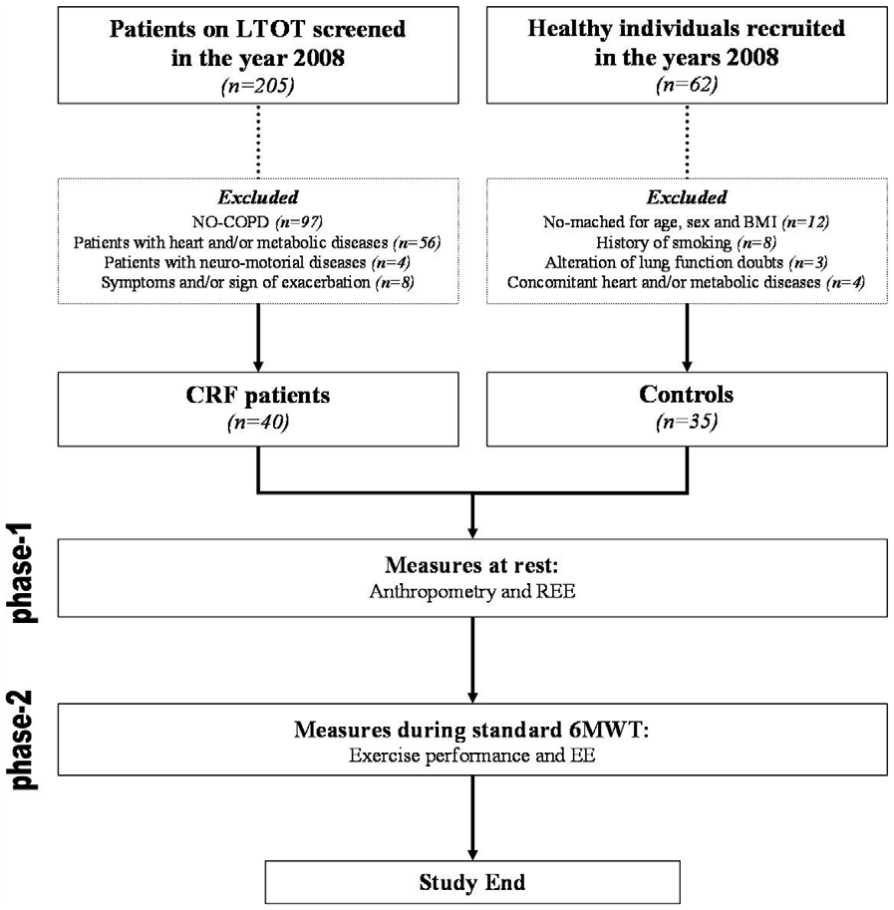
Study flow diagram.

The protocol for this trial and supporting CONSORT checklist are available as supporting information: see Figure 1.

### Objectives

In the present study we aimed at evaluating both 1) the validity of the SWA device and its reliability in terms of resting energy expenditure (REE), and 2) the effect of a walking aid in terms of EE to carry the oxygen canister in COPD patients with CRF.

### Patients and Controls

Consecutive patients fulfilling criteria for LTOT for at least 6 months and admitted to our rehabilitation center in the period January–December 2008 were screened. Among all of them, those without COPD and who reported any significant and clinically evident concomitant cardiovascular or metabolic disease were excluded. In addition, COPD with neuro-motorial diseases and/or cognitive failure unable to perform 6MWT were also excluded. Diagnosis and staging of COPD was made according to the GOLD definition and classification [13].

Healthy individuals matched by age, sex and body mass were selected in the same period and served as the control population. Those individuals reporting cardiovascular or metabolic disease, with a smoking history >5 pack/year, and/or with alterations at the spirometry were excluded. For details see Figure 1.

### Study protocol

This was a prospective two-phase study to compare data obtained in similar condition from both CRF patients and controls.

In *phase-1* we determined the validity of REE (calculated also by body weight-W correction) measured by SWA (REEa) in comparison with standard calorimetry (REEc). Measurements were taken on the study day in the morning while breathing room air in both groups.

In *phase-2* we then assessed EE and Metabolic Equivalents-METs by SWA in both groups during 6MWT performed in both modalities while breathing oxygen with a trolley carried on the dominant shoulder (*No-Aid*) *or* pulled on a wheeled cart (*Aid*) as previously described [12]. The weight of the oxygen trolley (3,5 kg) and the flow of oxygen administration during walking test were the same (3 litres/min) in both modalities and in the two study groups. The controls repeated the experiments while breathing room-air on a subsequent day.

The two walking modalities were conducted in random order on a same day (2 hour apart) and after achieving a complete recovery to baseline (dyspnoea and symptoms) in between. The same respiratory therapist unaware of the study purposes assisted all the tests.

### Measurements

#### General data

Demographic and anthropometric data were described in all the participants.

Body measures were collected by the same dietician and according to the Anthropometric Standardization Reference Manual [14] and the Italian Dietician National Association (ANDID) [15]. Body mass index (BMI) was calculated by dividing body weight (kg) for the squared height in meters (kg/m^2^). Body circumferences, obtained through a flexible and inextensible string at the level of both upper middle arm, waist and hip, were expressed in centimetres (cm). A mechanic plicometer (John Bull, British Indicators LTD) with standardized-pressure calibre was used at the level of triceps, biceps, under-scapular and up-iliac plica. A body bio-impedenziometric analyzer (Tanita Corporation BC-420MA) was used to assess body composition, respectively as fat (FMI) and fat free mass (FFMI) indexes and both expressed as kg/m^2^ [16].

Lung volumes (FEV_1_, FVC and FEV_1_/FVC) by means of a spirometer (Masterscope; Jaeger; Hoechberg, Germany) were expressed as absolute or percentage of predicted values [17]. Blood sample for the measurement of pH, PaO_2_, PaCO_2_, and PaO_2_/FiO_2_ ratio (Model 850; Chiron Diagnostics; Medfield, MA) was taken from the radial artery of CRF patients while in the sitting position and at rest.

The 6MWT was performed according to guidelines [18]. A pre-test evaluation, while wearing SWA, was executed in all the participants to limit the possible learning effect [19]. Patients showing >5% variation of EE recording by SWA during two pre-test 6MWT were not considered; the best of other two consecutive walked distance test was then selected for recording measures in the patients selected.

The total distance walked was calculated as absolute and percentage of predicted (% pred.) values in meters [20]. Oxygen saturation (SatO_2_) and maximal heart rate (HR max) were continuously monitored throughout the test by means of a pulsoximeter (Pulsox 3; Minolta; Tokyo, Japan). Baseline SatO_2_ (SaO_2_ pre-test), mean and minimal values of oxygen saturation (DeSaO_2_ and SaO_2-_nadir, respectively), rate of dyspnoea and muscle fatigue by modified Borg 10-points scale [21] at the beginning and the end of 6MWT, were also recorded.

#### Energy expenditure evaluation at rest and during walking test

The REEc and REEa recordings were obtained by the contemporary application of indirect calorimeter (Sensormedics VMAX Spectra 29N with canopy) and SWA (Pro2 Armband®, Body Media Inc, Pittsburgh, PA, USA) respectively. Tests were made at the same time in the morning, with empty stomach (at least 12 hours) and free from any type of strenuous physical activities for at least 24 hours [22].

In a room with fixed temperature between 70 and 75°F, the SWA was worn on the upper right arm of the participants while in orthostatic position at rest; this procedure was necessary for sensor skin temperature acclimatization. Pushing the button (timestamp) on SWA it was possible to identify exactly the periods of test's beginning-end (twenty minutes). The recorded period was multiplied by the value of 3 (determination of the REEa in one hour, kcal/h) and thereafter by 24 (REEa in a day, kcal/day) [23]. A software device (InnerView® Research Software, Professional version 6.1, USA) was then used for accurate reading and referring values.

During the indirect calorimetry test, oxygen-consumption (VO_2_) and volume of carbon dioxide (VCO_2_) were used in order to calculate the REEc in agreement with the formula of Weir [24]. The acceptability criteria of recordings as obtained from this test was a variation <5% in the respiratory-quotient/minute and VO_2_/min with at least 15 minutes of steady state.

Measurements during walking test were taken by SWA worn on the right upper arm and activated at least 10 minutes before starting the test: the timestamp signed exactly both beginning and end of walking time. Measures of change in EE and METs >5% obtained at 6MWT (pre-test and test) were excluded from data collection. Recordings of EE and METs were then normalized by walked meters ratio (EE/6MWT, kcal/mt and METs/6MWT, kcal/kg/h/mt) and used as study outcomes.

### Ethics

The Villa Pineta Foundation review board has approved the study which was conducted according to the Declaration of Helsinki. Participants gave their written informed consent to be included. No current external funding source has been assigned.

### Statistical analysis

Analysis was made by specific software (SPSS ver. 8.0 and Analyse-it® software Ltd. for Microsoft Excel standard edition). All the considered parameters were expressed as mean ± standard deviation (SD) and range. A probability value (p)<0.05 was considered as significant of difference for comparison.

The Bland and Altman [25] test was performed in order to evaluate repeatability and validity between different methods (VMAX Spectra 29N and SWA). With this analysis, repeatability can be accepted when no more than 5% of the calculated differences between values of each variable obtained during the two successive metabolic measurement exceeding the coefficient of repeatability, which is taken as twice the standard deviation of the differences between pairs of repeated observations.

Comparison of variables between groups and walking modalities (*No-Aid* and *Aid*) was carried out by *t*-test of Student and two ways ANOVA. The Wilcoxon and Kruskal-Wallis tests were also applied for non parametric variables.

In CRF population a sub-group comparison in patients with different performance at the 6MWT (< or ≥300 meters walked) was also made.

## Results

General characteristics of CRF patients and controls are reported in Table 1. COPD patients were in advanced stage of the disease with hypoxemia on ambient air at rest (data not displayed), but corrected with adequate oxygen supplement (mean need = FiO_2_ 28%) which approximates the severity of their CRF. All these patients were in GOLD stage IV by definition [13]. BMI were similar by definition between groups, although body circumferences and FFMI in male (17±2 and 19±1 kg/m^2^, respectively) were lower in patients than in controls.

**Table 1:** General characteristics of the population in study.

	CRF patients(n = 40)	Controls(n = 35)	p
Male/Female	26/14	17/18	0.123
Age, years	71.5 (7.3) [59–86]	69.4 (6.1) [60–83]	0.210
BMI, kg/m^2^	24.1 (4.2) [15.4–36.4]	25.8 (4.0) [19.4–31.6]	0.083
FEV_1_			
*L*	1.03 (0.3) [0.4–1.7]	2.71 (0.5) [2.0–3.6]	**<0.001**
*% predicted*	41.0 (9.6) [16–54]	115.6 (16.8) [87–148]	**<0.001**
FCV			
*L*	2.10 (0.6) [0.9–3.1]	3.38 (0.8) [2.1–5.0]	**<0.001**
*% predicted*	61.9 (12.2) [35–79]	118.7 (19.4) [80–156]	**<0.001**
FEV_1_/FCV, %	49.9 (12.0) [28–69]	76.8 (7.4) [69–88]	**<0.001**
PaO_2_, mmHg (on oxygen)	69.8 (11.5) [51–76]	-	-
PaCO_2_, mmHg (on oxygen)	46.2 (9.2) [31–53]	-	-
Supplemental oxygen required (FiO_2_), _%_	27.8 (3.2) [24]–[36]	-	-
PaO_2_/FiO_2_	254.3 (52.7) [162–357]	-	-
Arm circumference, cm	25.9 (2.7) [21]–[35]	28.4 (3.1) [24]–[35]	**0.009**
Waist circumference, cm	84.6 (8.0) [72–99]	97.0 (13.7) [70–125]	**<0.001**
Hip circumference, cm	88.6 (7.5) [74–101]	99.8 (5.7) [91–111]	**<0.001**
Tricipital plica, mm	8.8 (3.2) [1.1–14.3]	8.8 (8.8) [1.1–25]	0.635
Bicipital plica, mm	8.7 (7.0) [1.1–25]	8.2 (9.0) [1.1–30]	0.822
Sub-scapular plica, mm	12.3 (6.8) [1.1–25]	17.4 (24.4) [1.1–65]	0.264
Up-iliac plica, mm	14.8 (6.3) [1.1–29]	22.5 (26.5) [1.1–58]	0.115
FMI, kg/m^2^			
*Male*	6.30 (3.7) [0.9–10.4]	8.26 (2.0) [5.7–11.0]	0.241
*Female*	9.55 (3.0) [5.6–13.4]	10.04 (4.3) [5.7–20.7]	0.779
FFMI, kg/m^2^			
*Male*	17.01 (2.1) [13.2–19.8]	19.42 (1.3) [17.5–21.0]	**0.022**
*Female*	15.07 (1.6) [13.4–18.1]	16.04 (1.9) [13.6–19.1]	0.258

Value expressed as mean ± standard deviation (SD) and [range].

FMI: Fat mass index; FFMI: Fat-free mass index.

### Phase-1

**Figure 2:**
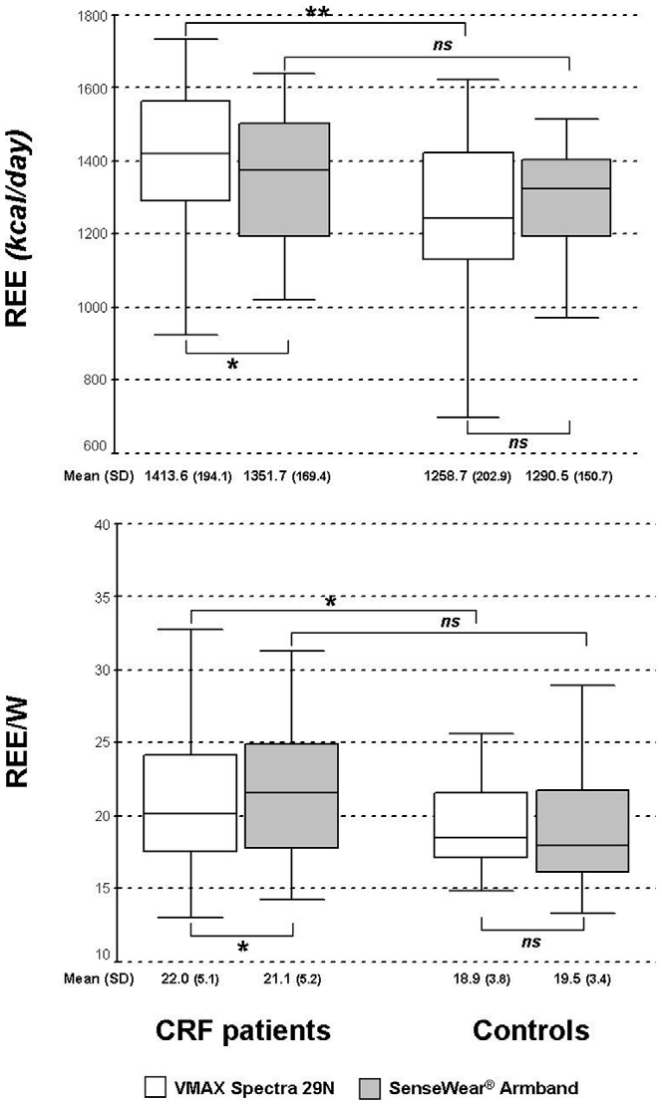
Comparison of REE and REE/W values obtained by indirect calorimetry and SWA in the two study groups (for details see Methods). *p<0.05, **p<0.001, *ns: not significant*.

Whole comparison of REEc and REEa in the study groups is shown in Figure 2. As expected, mean data were higher in CRF with a significant difference for REEc only (1413 *vs* 1258 kcal/day, in patients and controls, respectively). Similar findings were shown by analysis of REE and W ratio (REE/W).

**Figure 3:**
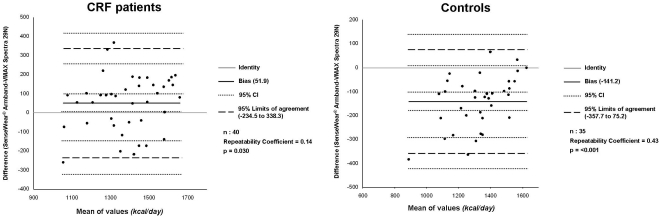
Bland & Altman analysis and repeatability coefficients in the two study groups.

SWA assessment significantly underestimated REEa when compared with standard REEc in CRF but not in healthy population. The Bland and Altman analysis between the two methods (Figure 3) has shown a greater repeatability grade in controls than in CRF patients (0.43 and 0.14, respectively).

### Phase-2

Table 2 summarizes results of the 6MWT as obtained in the two groups and comparing both modalities of assisted (*Aid*) and unassisted (*No-Aid*) walking. Exercise performance in both modalities was lower in CRF patients when compared with healthy individuals. However, at difference with controls, CRF experienced a significant improvement with *Aid* as compared with *No-Aid* in terms of meters walked (+23,5 m) and perceived symptoms (−0,86 and −0,95 at the Borg dyspnea and leg fatigue, respectively).

**Table 2:** Results of the exercise performance in the population in study.

	CRF patients#(n = 40)			Controls#(n = 35)			Controls in room air(n = 35)		
	*No-Aid*	*Aid*	p	*No-Aid*	*Aid*	p	*No-Aid*	*Aid*	p
Distance walked at 6MWT, meters	295.2 (90.8)	318.7 (90.0)	**0.003**	506.2 (50.5)	482.1 (43.6)	**<0.001**	501.0 (54.4)	484.2 (43.5)	**0.002**
Distance walked at 6MWT, % predicted	61.4 (17.8)	67.5 (19.0)	**0.003**	110.9 (14.1)	103.9 (13.8)	**<0.001**	109.1 (14.9)	105.2 (13.4)	**<0.001**
Peak effort dyspnoea, Borg†	4.67 (2.0)	3.81(1.8)	**<0.001**	1.28 (0.7)	1.51 (0.7)	0.053	1.14 (0.7)	1.48 (0.7)	**0.005**
Peak leg effort fatigue, Borg†	3.60 (1.8)	2.65 (1.2)	**<0.001**	1.40 (0.7)	1.59 (0.7)	0.076	1.31 (0.9)	1.53 (0.7)	**0.024**
RR at peak effort, breaths/min	22.8 (4.1)	21.6 (3.2)	**<0.001**	18.7 (1.7)	19.1 (2.0)	0.051	18.7 (2.3)	19.4 (2.2)	**0.001**
HR max, beats/min	109.2 (9.9)	106.1 (11.5)	0.067	105.4 (9.2)	105.0 (11.4)	0.751	106.5 (10.2)	110.6 (10.7)	**0.036**
SaO_2_ pre-test, %	94.1 (2.3)	94.8 (1.8)	0.601	97.4 (1.0)	97.6 (0.8)	0.905	95.6 (1.3)	95.7 (1.3)	0.231
SaO_2_, %	88.7 (3.3)	89.4 (3.8)	0.060	95.5 (1.1)	95.4 (0.9)	0.497	94.0 (1.1)	93.5 (1.2)	**0.001**
Reduction of SaO_2_ during 6MWT, %	5.35 (2.8)	5.36 (3.6)	0.990	1.86 (1.1)	2.24 (1.0)	**0.049**	1.62 (1.0)	2.20 (1.2)	**<0.001**
Nadir of SaO_2_ during 6MWT, %	84.1 (5.9)	85.5 (5.8)	**0.019**	93.7 (1.5)	93.7 (1.8)	0.926	91.4 (1.9)	91.6 (1.8)	0.546

Value expressed as mean ± SD.

# Test performed breathing oxygen at 3 L/min.

† Differences calculated using no-parametric test of Kruskal-Wallis and Wilcoxon test.

**Figure 4:**
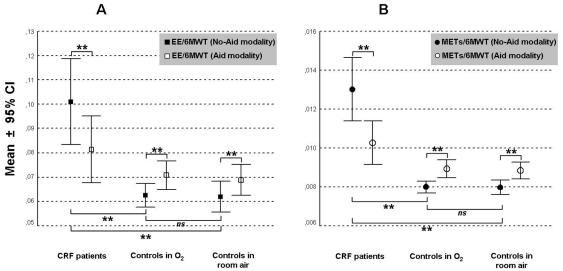
Comparison of EE outcomes recorded during both assisted (*Aid*) and unassisted (No-*Aid*) walking in both CRF patients (*panel A*) and Controls (*panel B*) (for details see Methods). **p<0.001, *ns: not significant*.

Metabolic data as obtained in the two study groups during walking tests (EE/6MWT and METs/6MWT) are displayed in Figure 4. Walking energy expenditure was higher in CRF than in controls and in both walking modalities. Interestingly, this was significantly lowered with *Aid* modality in CRF only (−0,19 kcal/mt at EE/6MWT and −0,003 kcal/kg/h/mt at METs/6MWT). An opposite behaviour was found in the control situation, independent on the oxygen breathing.

It is also interesting to note that, when comparing the *Aid* and *No-Aid* modalities, given a similar improvement of meters walked (mean difference +28±34 and +19±58 m, respectively-ns) and symptoms, the magnitude of reduction in walking energy expenditure with *Aid* was higher in the sub-group of CRF patients performing <300 m (mean difference −0,028±0,02 and −0,0041±0,003 for EE/6MWT and METs/6MWT, respectively-p<0.001) than in those walking ≥300 m (mean difference −0,010±0,01 and −0,0013±0,001 for EE/6MWT and METs/6MWT, respectively-p<0.05) at baseline 6MWT (data not showed in table).

## Discussion

Present study provides additional information about SWA device as a feasible and valid instrument to assess energy expenditure in disabled CRF patients fulfilling the criteria for and using LTOT. Measurement of EE by means of SWA demonstrated that assisted walking results in a substantial saving when compared with conventional walking in this population.

The use of complex methods to assess EE in humans (i.e. radio-isotopic systems), although theoretically ideal, is rather difficult due to several technical problems, elevated costs and the inability to objectively record specific patterns during physical activity. On the other hand, easy devices like pedometers, although optimally validated during walking in healthy subjects [26], are not adequate to assess metabolic data, nor they may be sensible enough in case of slow body movements, such as the case of the typical walking of disabled COPD patients [27].

In recent years, multi-axial accelerometers and integrated body-monitors with skin sensors (such as SWA) have been tested for their validity and reliability to assess EE in healthy subjects [28] [29], in COPD [30] and in other chronic conditions [7] [8] [31].

Validity of SWA, compared with indirect calorimetry as the *gold standard* method, has been already and previously demonstrated in healthy individuals and in patients with extra-pulmonary disorders [1]–[8]. In COPD patients, SWA applied during standard 6MWT demonstrates a metabolic accuracy equivalent to the portable “breath-to-breath” system with a under-estimation of approximately 1% when the walking speed was <3 miles/hours [9]. More recently Hill and coworkers [10] have found a good coefficient of repeatability with SWA, when compared with indirect calorimetry, when measuring energy expenditure in a population of COPD of similar degree of lung obstruction as in our present research, but without respiratory failure.

In our study, REE level with both methods was considerably more elevated in CRF patients than in healthy controls, which clearly stands for the increased metabolism at rest in these patients [32] [33]. However, SWA assessment underestimated REE value in COPD but not in controls, which confirms previous separate findings in the same disease [10] and in normal-weight individuals [1] [2].

This powerlessness of SWA could be linked to the technical characteristics of the device in data recording. Indeed, skin sensors of SWA prevent from perceiving both the pulmonary and respiratory muscle activity quota, which is known to be increased in CRF patients [34] [35]. To confirm the lack of full repeatability between SWA and indirect calorimetry, the Bland and Altman coefficient has shown a low degree of repeatability, which was lower in CRF patients than in controls (see Figure 3) with 2 patients (5% of total) out of the 95% limits of agreement (between −234 and +338). This result in CRF seems to be worse than the previous one [10]; however, our patients are least comparable with those in that study due to their different clinical characteristics and overall severity mainly related with the use of LTOT, and with the specific reduction in FFMI (at least in male subjects, as shown in Table 1) [16] [36] [37]. Moreover and finally, repeatability coefficient (0.43) was also lower in our controls when compared with those in previous study [2]; this aspect is probably linked to the older age of our individuals thus influencing metabolic recordings with SWA, to our knowledge not still validated in elderly population.

In phase-2 of our study we then principally aimed at assessing EE by SWA during assisted or unassisted walking, in order to confirm the potential energy saving we have previously hypothesized when using the trolley on a wheeled cart to facilitate ambulation of CRF on domiciliary oxygen [12]. The usefulness and reliability of EE assessment by SWA during flat walking has been recently confirmed in the population of COPD [10].

Present study documented that aided modality of walking enabled these severe COPD patients to considerably save energy, which was not the case in the control population. In particular, due to the weight reduction of the oxygen trolley put on the shoulder, the aided modality to walk allowed patients to have a meaningful reduction of energy during the test both in terms of expenditure (about 20% less) and METs (about 25% less). Moreover and interestingly, this metabolic together with the clinical benefit in the *Aid* modality is much higher in the subgroup of patients at the lowest grade of exercise performance (<300 m. at 6MWT, 50% of the our sample).

The practical translation of this finding is that any form of aid might thus increase physical activity in this very advanced and disabled population [38] [39]. Indeed, improvement of the metabolic work would parallel the individual's reserve in heart rate and oxygen consumption, directly linked with any positive change in the aerobic activity [38]. Notwithstanding, this result only leads to hypothesize a bio-enzymatic adaptation at the skeletal muscle level, since our study was not designed as physiologic.

The lack of positive change with EE and symptoms during assisted walking in the controls would be more likely related to a sort of “obstacle-effect” when using the aid device. In this population, pulling the wheeled cart on the floor translated in additional work, symptoms and energy expenditure; similar (and even worse) results were found when breathing room air, thus excluding the possible bias effect of oxygen inhalation on EE measurement.

Taking these consideration all together, we conclude that SWA can be a feasible and valid device to measure and to monitor energy expenditure in very severe COPD patients with chronic respiratory failure. In these disabled patients the daily use of ambulatory oxygen can be helped by a simple and small wheeled cart with is associated by a better performance, reduced symptoms and relevant saving in energy consumption, which is more likely to observe in those patients with lower walking performance (<300 m at the 6MWT).

### Limitations

Even if the SWA device has been validated in healthy and COPD patients showing to have an elevated reliability during 6MWT, we can't exclude that the energy saving during a walking aided may be a reflection of the inability of the SWA to capture EE when the upper arm is not moving normally, due to the pulling of the walking aid.

Moreover, one could criticize the choice of 6MWT as the best test to assess EE in healthy individuals. In general, this is certainly a test at which work rate is not standardized; however, the application of clear and standardized instructions during assessment translates into a likely ability of the individuals to walk at his/her best intensity, which is known to be reached within 2 minutes after beginning. Therefore, the standard walking is a widely accepted and reliable test to assess physical performance even in normals [40].

Finally, and despite the criteria adopted to select our study population (see Methods), a substantial number of COPD still have concomitant cardiovascular and metabolic diseases at least at a sub-clinical level that might theoretically bias the sample selection.

Taking the limits and/or advantages of SWA device, it is likely that future studies will clarify its role as a metabolic holter in elderly population, in chronic respiratory diseases other than COPD (i.e. pulmonary fibrosis), and in diseases with skin involvement (i.e. sclerodermia).
